# Changes and Correlation Between Physiological Characteristics of *Rhododendron simsii* and Soil Microbial Communities Under Heat Stress

**DOI:** 10.3389/fpls.2022.950947

**Published:** 2022-07-22

**Authors:** Lei Liu, Wei Lin, Li Zhang, Xuexiao Tang, Yue Liu, Siren Lan, Shusheng Wang, Yan Zhou, Xiaochou Chen, Ling Wang, Xiang Chen, Lijin Guo

**Affiliations:** ^1^Key Laboratory of Genetics and Germplasm Innovation of Tropical Special Forest Trees and Ornamental Plants, Ministry of Education/College of Forestry, Hainan University, Haikou, China; ^2^College of Forestry, Fujian Agriculture and Forestry University, Fuzhou, China; ^3^College of Tropical Crops, Hainan University, Haikou, China; ^4^Lushan Botanical Garden, Jiangxi Province and Chinese Academy of Sciences, Lushan, China; ^5^Guizhou Botanical Garden, Guiyang, China; ^6^Fuzhou Qinting Lake Park Management Office, Fuzhou, China; ^7^Institute of Biology, Guizhou Academy of Sciences, Guiyang, China

**Keywords:** heat stress, physiological characteristics, *Rhododendron simsii*, sequencing, soil microorganisms

## Abstract

The relationship between *Rhododendron simsii* and its soil microbial community under heat stress was not clear. In this study, the effects of heat stress on the physiological characteristics, soil physicochemical properties and soil microbial community structure of *R. simsii* were investigated. The experimental control (CK) was set as day/night (14/10 h) 25/20°C and experimental treatments were set as light heat stress (LHS) 35/30°C and high heat stress (HHS) 40/35°C. Our results showed that, compared with CK, LHS treatment significantly increased malondialdehyde, hydrogen peroxide, proline and soluble sugar contents, as well as catalase and peroxidase activities, while HHS treatment significantly increased ascorbate peroxidase activity and decreased chlorophyll content. Compared with CK, LHS treatment significantly reduced soil ammonium-nitrogen and nitrate-nitrogen content, while HHS significantly increased soil ammonium-nitrogen content. Compared with CK, both treatments changed the soil microbial community structure. For bacterial community, LHS and HHS treatment resulting in the significant enrichment of *Burkholderia-Caballeronia-Paraburkholderia* and *Occallatibacte*, respectively. For fungal community, LHS treatment resulting in the significant enrichment of *Candida, Mortierella* and *Boothiomyces*. The redundancy analysis showed that plant physiological characteristics, soil ammonium-nitrogen content were significantly correlated with the soil microbial community. Therefore, heat stress altered the soil microbial community structure, and affected the availability of soil available nitrogen, which in turn affected the physiological characteristics of *R. simsii*. We suggest that soil microbial community may play an important role in plant resistance to heat stress, and its mechanism deserves further study.

## 1. Introduction

*Rhododendron* is a genus of plants in the family of Ericaceae, which is known as the “beauty flower” with high ornamental, ecological and medicinal value. A total of 571 species of *Rhododendron* were found in China, accounting for about 55% of the total number of *Rhododendron* in the world (Yu et al., 2017). The genus *Rhododendron* is mainly distributed in high-altitude areas in the southwest China and grow in a cold environment with poor heat resistance (Shrestha et al., 2018). The development of the *Rhododendron* industry in China is slow, which is primarily caused by insufficient heat resistance of *Rhododendron* (Li et al., 2020). Therefore, it is of great significance to study the response mechanism of *Rhododendron* to heat stress.

Different kinds of abiotic stresses such as salinity, drought, cold, and heat stresses can be regarded as the most destructive sorts of stressors, which extremely affect physiological and biochemical characteristics in plants (Goharrizi et al., 2019, 2020, 2021; Huo et al., 2021). Under heat stress, excessive reactive oxygen species (ROS) are produced in plant cells, which in turn can cause oxidative stress, and result of damage of plant cells (Duc et al., 2018). Under heat stress, plants automatically perform mechanism such as regulation of osmotic substance concentration, production of antioxidants and chaperone signal transduction and transcriptional activation to regulate water balance and scavenge excess reactive oxygen species in plants, which protect the plant cell membrane (Pei et al., 2021). In recent years some studies reported the effects of heat stress on physiological characteristics, such as malondialdehyde (MDA), antioxidant enzyme activities and photosynthetic properties of *Rhododendron* (Shen et al., 2017; Zhao et al., 2018; Yang et al., 2020). In addition, some studies suggested that some measures can increase the heat resistance of *Rhododendron*, such as pretreated *Rhododendron* by applying external ethylene (Zhao et al., 2022), hydrogen peroxide (H_2_O_2_) (Geng et al., 2019). However, the response mechanism of *Rhododendron* to heat stress is still unclear.

Heat stress can alter the availability of soil nutrients, which in turn affect the nutrient absorption of plants (Hussain et al., 2019). Jian et al. (2020) found that the carbon and nitrogen content of agricultural soil was closely related to the average annual temperature. Wang et al. (2019) found that heat stress had significant effects on the availability of soil carbon and nitrogen sources, and soil enzyme activities. Moreover, heat stress significantly reduced the availability of soil nutrient elements, leading to a reduction in plant biomass (Yeasmin et al., 2019). Mu et al. (2021) reported that inoculation of ericoid mycorrhizae fungi can improve the heat and drought tolerance of plants and alleviate the damage of *Rhododendron* caused by heat stress. Therefore, plant growth and development under heat stress are closely related to soil nutrients, soil enzyme activities and other soil physicochemical properties, and soil microorganisms may also play an important role in the process of plant response to heat stress.

Soil microorganisms are important drivers of soil nutrient cycling and have a major impact on the availability of soil organic carbon and nutrients element (Hallama et al., 2019). As a life element of plants, nitrogen is important for plant growth and development, and is involved in the synthesis of proteins and nucleic acids as well as a range of enzymes (Thirkell et al., 2016). Veresoglou et al. (2012) reviewed the mechanisms of soil fungi affecting soil nitrogen cycling, and indicated that soil nitrogen cycle is basically driven by microorganisms. For example, as the rate-limiting step of the nitrogen cycle, nitrification is started with NH${}_{4}^{\;+}$ and ended with NO${}_{3}^{\;-}$, respectively, and is mainly driven by ammonia-oxidizing bacteria and ammonia-oxidizing archaea (Veresoglou et al., 2019). However, plants and soil microbes may also compare for nitrogen under certain conditions, such as heat stress (Veresoglou et al., 2012). Previous studies have only focused on the effects of heat stress on plant physiological characteristics, and growth and development of plant, while few studies have focused on the relationship among soil microbial communities, plant physiological characteristics and plant heat resistant (Wang et al., 2019; Yeasmin et al., 2019; Mu et al., 2021). Therefore, it is essential to study the effects of heat stress on soil microbial communities, heat tolerance of plants, and the relationship between them.

We studied the effect of heat stress on physiological characteristics of *Rhododendron*, soil physicochemical properties and soil microbial communities. This study aimed to reveal the response mechanisms of the interaction between *Rhododendron* and soil microorganisms to heat stress. We hypothesized that soil microbial community plays an important role in *Rhododendron* resistance to heat stress.

## 2. Materials and Methods

*Rhododendron simsii* was selected as the test material for this study. One-year-old *R.simsii* seedlings were selected, and they were provided by Jianhui Seedlings Co. Ltd., Zhangzhou, Fujian Province, China. The average height of the seedlings was 20 cm, and they were planted in plastic pots of 12.5 cm in height, 14.5 cm in upper diameter and 10 cm in lower diameter, and each pot was filled with 1.5 kg culture substrate. The culture substrate was Klasmann Peat: perlite = 3:1 (v/v), and the soil pH was 4.65. *R. simsii* were grown in artificial climate chambers with conditions as follows: (1) day/night: 14/10 h, (2) light intensity of 2,000 lx, and (3) a relative humidity of 70–80%. Plants were precultured for 1 month before applying experimental treatment.

### 2.1. Experimental Design

**Figure 1:**
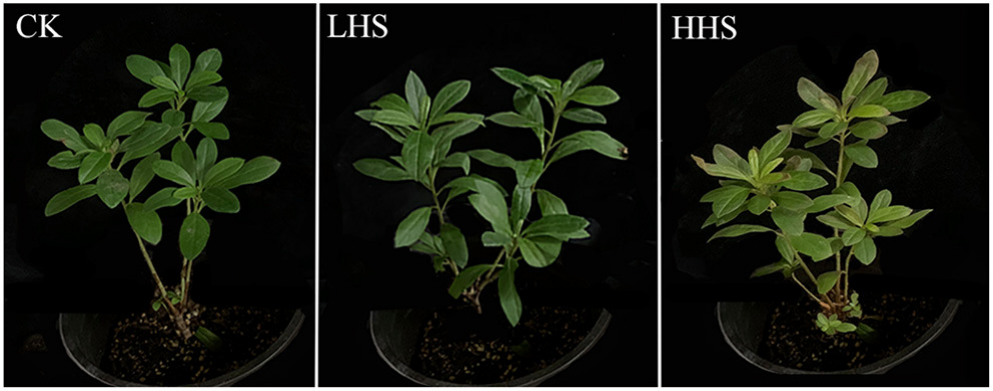
Plants condition after 6 days of different temperature treatment. CK, control (25/20°C); LHS, light heat stress (35/30°C); HHS, high heat stress (40/35°C).

The experiment was conducted from June to August 2021 at the Key Laboratory of Genetics and Germplasm Innovation of Tropical Special Forest Trees and Ornamental Plants (Hainan University), Ministry of Education. A completely randomized design was used in this study. Temperature treatment was conducted using artificial climate chamber. The experimental control (CK) and heat stress treatments were described as follow: (1) CK (day/night: 14/10 h) 25/20°C, (2) light heat stress (LHS) 35/30°C, (3) high heat stress (HHS) 40/35°C, respectively, with four replications. To avoid water stress due to heat stress, trays were placed at the bottom of the pots with a water level of 1 cm in the trays during experimental treatment. Other environmental conditions and management measures of experimental groups were consistent with those of the control group. After 6 days of heat stress, we collected the plants and soil samples. The plant situation of each treatment is shown in Figure 1. Some leaves were used for measuring plant physiological and biochemical characteristics. After collecting the plant samples, the bulk soil samples were mixed evenly with the quartering method and were sifted through a 2 mm sieve. Then part of the bulk soil samples was frozen in the −20°C refrigerator for the determination of the soil physical and chemical properties, while the other samples was frozen in sterile 10 mL centrifuge tubes with liquid nitrogen and placed in a −80°C refrigerator for the determination of the microbial community structure.

### 2.2. Indicator Measurement and Methods

#### 2.2.1. Measurement of Plant Physiological Indexes

MDA content (nmol g^−1^), H_2_O_2_ content (μmol g^−1^), proline (Pro) content (μg g^−1^), soluble sugar content (mg g^−1^), chlorophyll content (mg g^−1^), catalase (CAT) activity (U g^−1^), peroxidase (POD) activity (U g^−1^), and ascorbate peroxidase (APX) activity (U g^−1^) were determined using Solarbio detection kits (Beijing Solarbio Science and Technology Co., Ltd.). The soluble protein content (μg g^−1^) was determined using the Jiangsu Jingmei kit (Jiangsu Jingmei Biological Technology Co., Ltd.). Refer to the kit instructions for specific methods. All measurements were carried out with a UV-Visible spectrophotometer (UV-5500, Shanghai Yuan analysis, China).

#### 2.2.2. Determination of Physical and Chemical Properties of Soils

The soil pH was determined by a pH meter (PHS-3E, Shanghai Keyou, China), and the soil and water mass ratio of soil suspension was 1:5. Soil ammonium-nitrogen was determined using the indophenol blue colorimetric method, and nitrate-nitrogen was determined using the UV spectrophotometric method, both were measured using a UV-Visible spectrophotometer (UV-5500, Shanghai Yuan analysis, China). Soil water-soluble organic carbon (WDOC) was measured by the dichromate external heating method and soil microbial nitrogen (MBN) was measured by the chloroform fumigation incubation method (Bao, 2000).

#### 2.2.3. Soil Microbial Community Sequencing

Soil sample per replicate was stored in dry ice and sent to Personal Biotechnology Co., Ltd. (Shanghai, China) for determining soil microbial community using high throughput sequencing. Total genomic DNA samples were extracted using the OMEGA Soil DNA Kit (M5635-02) (Omega Bio-Tek, Norcross, GA, USA). The quantity and quality of extracted DNA were determined using a NanoDrop NC2000 spectrophotometer (Thermo Fisher Scientific, Waltham, MA, USA) and agarose gel electrophoresis, respectively. Illumina Novaseq sequencing platform was used for amplicon sequencing and library construction of the ITS 1 region of fungal ITS rDNA and the V3-V4 region of bacterial 16 S rDNA. The fungal sequencing primers were TIS 5 (5′-GGAAGTAAAAGTCGTAACAAGG-3′) and ITS 2 (5′-GCTGCGTTCTTCATCGATGC-3′), and the bacterial sequencing primers were 338 F (5′-ACTCCTACGGGAGGCAGCA-3′) and 806 R (5′-GGACTACHVGGGTWTCTAAT-3′) (Xu et al., 2018). The sequences were filtering and quality assessment was done by DADA2 method of QIIME2 software (2019.4, https://docs.qiime2.org/2019.4/tutorials/) after consolidation of the original data, and then high-quality reads were divided into operational taxonomic units (OTUs) with 97% similarity.

### 2.3. Data Analysis

All data were sorted out by Excel 2020. Data on plant physiological characteristics and soil physicochemical properties were expressed as mean ± standard deviation. SPSS 17.0 was used for one-way analysis of variance, and the Duncan test was used for significance analysis. Figures were plotted using Prism 8.0. Redundancy analysis (RDA) was analyzed and mapped using the Vegan package in *R*, based on soil microbial communities, plant physiological characteristics, and soil physicochemical properties.The linear discriminant analysis coupled with effect size analysis (LEfSe) was employed to explore statistically different biomarkers between treatments (Wu et al., 2022).

## 3. Results

### 3.1. Effect of Heat Stress on MDA and H_2_O_2_ in *R. simsii*

**Figure 2:**
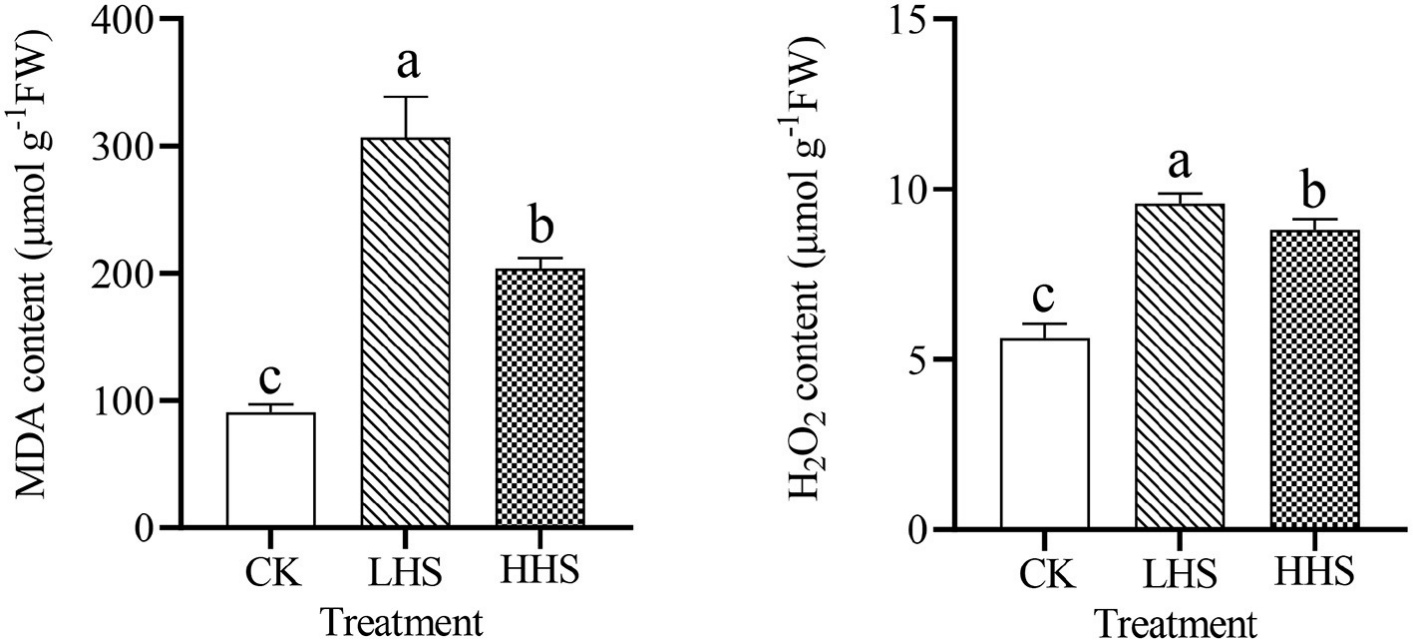
Contents of malondialdehyde (MDA), contents of hydrogen peroxide (H_2_O_2_) in *Rhododendron simsii*. leaves under CK, control (25/20°C); LHS, light heat stress (35/30°C); HHS, high heat stress (40/35°C). Each value represents the mean ± standard deviation (*n* = 4). Different lowercase letters indicate the significant differences (*P* < 0.05) among treatment by Duncan test.

As shown in Figure 2, heat stress significantly increased MDA content. Compared to CK, LHS and HHS treatments significantly increased MDA content by 239.0 and 125.3% (*P* < 0.05), respectively. Meanwhile, MDA content tended to increase firstly and then decrease with the increase of treatment temperature. Compared to LHS treatment, HHS treatment decreased MDA content by 33.5% (*P* < 0.05). Compared to CK, LHS, and HHS treatments significantly increased H_2_O_2_ content by 70.0% and 56.3% (*P* < 0.05), respectively. Similarly, the H_2_O_2_ content also tended to rise and then decrease under increasing heat stress. Compared to LHS treatment, HHS treatment decreased H_2_O_2_ content by 7.9% (*P* < 0.05).

### 3.2. Effect of Heat Stress on Osmoregulation, Enzyme Activity, and Chlorophyll Content in *R. simsii*

**Figure 3:**
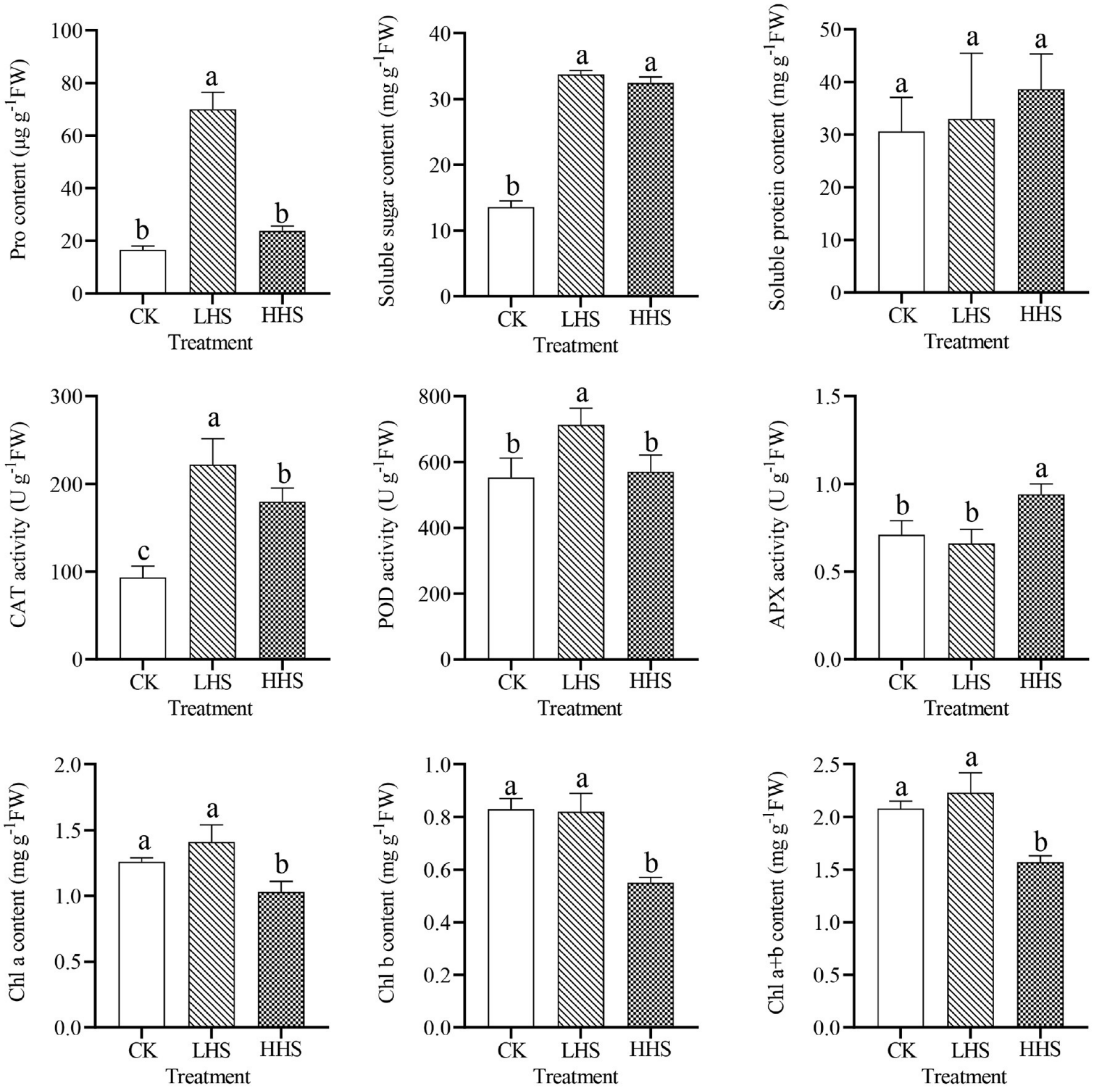
Effect of heat stress on physiological indexes of leaves in *Rhododendron simsii*. Each value represents the mean ± standard deviation (*n* = 4). Different lowercase letters indicate the significant differences. CK, control (25/20°C); LHS, light heat stress (35/30°C); HHS, high heat stress (40/35°C); Pro, proline; CAT, catalase; POD, peroxidase; APX, ascorbate peroxidase; Chl *a*, chlorophyll *a*; Chl *b*, chlorophyll *b*; Chl *a* + *b*, chlorophyll *a* + *b*.

As shown in Figure 3, LHS treatment significantly increased the content of Pro and soluble sugar by 324.1 and 148.7% (*P* < 0.05), respectively. Compared to CK, HHS treatment significantly increased the soluble sugar content by 139.2% (*P* < 0.05) but had no significant effect on the Pro content. The soluble protein content gradually increased with heat stress, but there was no significant difference in different treatments, which means in the present study, with the increase in heat stress degree, the activity of CAT and POD in *R. simsii* leaves tended to increase first and then decrease, while the activity of APX tended to decrease first and then increase. Compared to CK, LHS, and HHS treatments increased CAT activity by 137.9 and 92.7% (*P* < 0.05), respectively. Compared to CK, LHS treatment significantly increased POD activity by 29.0% (*P* < 0.05), but HHS treatment had no significant effect on POD activity. In addition, there was no substantial change in APX activity under LHS treatment compared to CK, but HHS treatment significantly increased APX activity in leaves by 28.2% (*P* < 0.05). The result showed that there was no significant change in chlorophyll content under LHS treatment compared to CK (*P*>0.05), but HHS treatment significantly decreased *R. simsii* chlorophyll *a* content by 22.3%, chlorophyll *b* content by 50.9%, and chlorophyll *a*+*b* content by 32.5% (*P* < 0.05).

### 3.3. Effect of Heat stress on Soil Physicochemical Properties

Compared with CK, LHS treatment significantly reduced the content of ammonium-nitrogen by 23.7%, and HHS treatment significantly increased ammonium-nitrogen by 16.8% (*P* < 0.05). Compared with CK, LHS and HHS reduced nitrate nitrogen-content by 28.0 and 31.4% (*P* < 0.05), respectively. In comparison with CK, soil pH was reduced by 3.3% in LHS treatment, soil WDOC and MBN under two treatments were lower, while none of them were significant (Table 1).

**Table 1:** pH and nutrient contents of soil at different treatments.

**Treatment**	**pH-value**	**WDOC content (g kg^**−**^^**1**^)**	**$NO_{3}^{\;-}$−N content (mg kg^**−1**^)**	**$NH_{4}^{\;+}$−N conten (mg kg^**−1**^)**	**MBN content (mg kg^**−1**^)**
CK	4.25 ± 0.07a	7.27 ± 0.85a	8.61 ± 0.46a	280.05 ± 10.04b	23.94 ± 1.95a
LHS	4.14 ± 0.08a	7.03 ± 0.38a	6.73 ± 0.54b	226.40 ± 11.39c	20.76 ± 3.60a
HHS	4.26 ± 0.08a	6.96 ± 0.64a	6.55 ± 0.63b	327.06 ± 37.66a	21.82 ± 1.86a

*Data (means ± SDs) are given for experimental treatments (n = 4) and different lowercase letters denote significant differences among treatments at P < 0.05. CK, control (25/20°C); LHS, light heat stress (35/30°C); HHS, high heat stress (40/35°C); WDOC, water dissolved organic carbon; NO${}_{3}^{\;-}$−N, nitrate-nitrogen; NH${}_{4}^{\;+}$−N, ammonium-nitrogen; MBN, microbial nitrogen*.

### 3.4. Response of Bacterial Community

**Figure 4:**
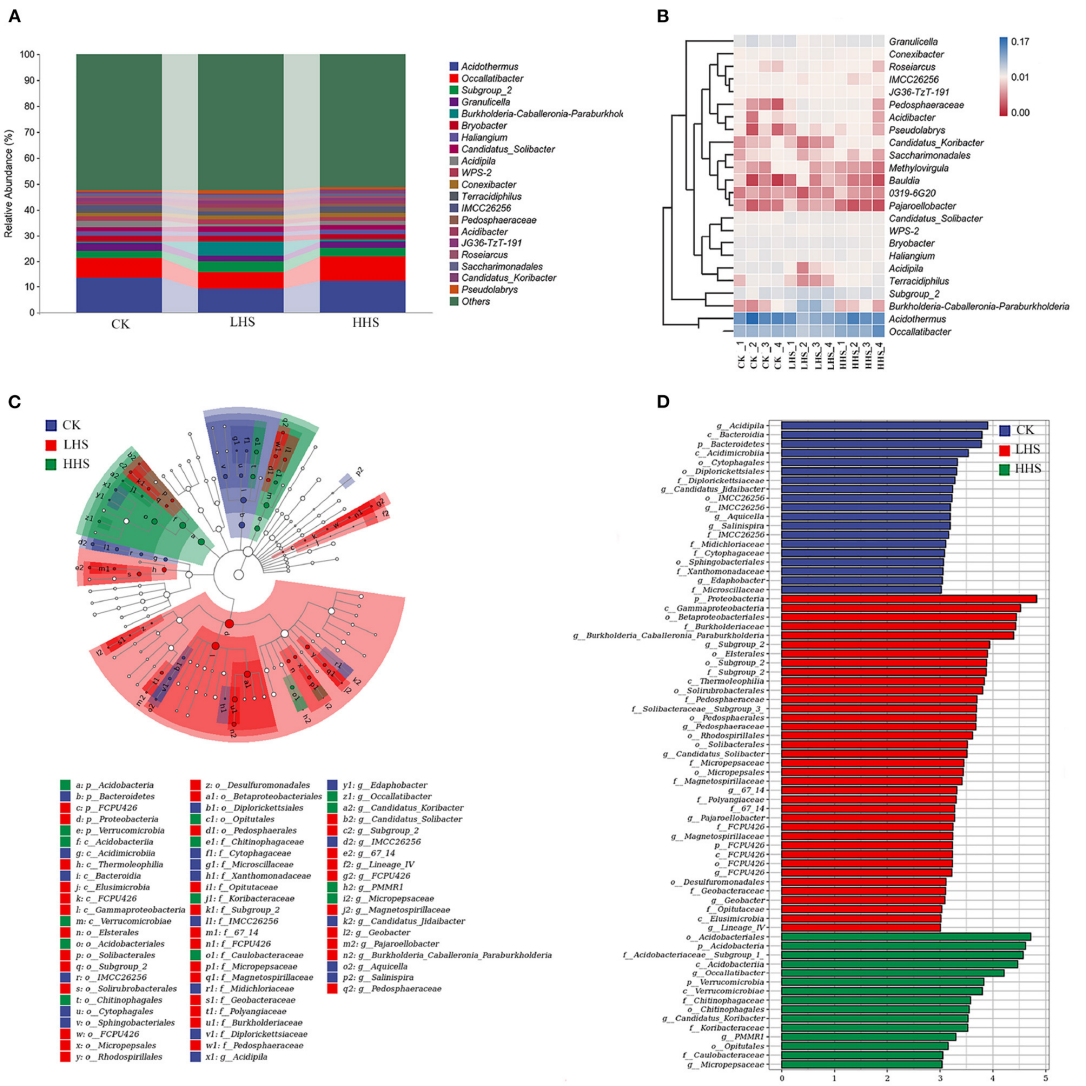
Comparison of bacterial communities in CK, LHS and HHS samples. **(A)** Bacterial taxa at genus level. **(B)** Heatmap showing changes in microbial community on genus level. **(C)** Cladograms indicate the phylogenetic distribution of microbial lineages associated with the three treatments. Each circle diameter is proportional to the taxon abundance. The strategy of multiclass analysis is strict (all classes differential). Circles represent phylogenetic levels from Kingdom to genus. **(D)** LDA bars indicate microbial groups within three treatments with LDA score higher than 3.0. The difference in abundant clades of each treatment are represented by colors as in cladograms, and the LDA scores of these clades indicate the degree of statistically and biologically difference. CK, control (25/20°C); LHS, light heat stress (35/30°C); HHS, high heat stress (40/35°C); p, phylum; o, order; c, class; f, family; g, genus.

A total of 891,231 validated bacterial sequences were obtained after passing quality filtering. Sequence reading for per sample ranges from 67,100 to 114,651. We detected a total of 490 genera, and the total abundance of OTUs was higher in the LHS treatment than that in the other two treatments (Supplementary Figure 1). At the genus level, there were 24 genera with relative abundances higher than 1%, and the three most abundant *Acidothermus* was dominant representing 5.4–7.9% of the bacteria in all treatments, followed by the *Occallatibacter* and the Subgroup_2 (Figure 4A and Supplementary Figure 1). Compared to CK, the abundance of *Acidothermus* decreased by 32.2%, while the abundance of *Burkholderia-Caballeronia-Paraburkholderia* (BCP) increased by 573.5% in LHS treatment. Compared to CK, the abundance of *Occallatibacter* increased by 18.0% in HHS treatment (Figure 4B and Supplementary Figure 1). The sequencing covered a substantial portion of the bacterial diversity because the rarefaction curves tended to be plain (Supplementary Figure 3A). Alpha diversity including Simpson index, Shannon index, Chao1 index, and Observed-species did not show a significant difference with different treatments (*P*>0.05) (Supplementary Figure 4A). Furthermore, Cladograms (Figure 4C) and LDA (Figure 4D) of LEfSe analysis were used to reveal key biomarkers of different groups. More bacterial taxa were detected in LHS treatment (36 clades, 2 phylum, 4 classes, 10 orders, 10 families and 10 genera) than in other treatments. At the genus level, *Acidipila, Candidatus_Jidaibacter, Aquicella, Salinispira*, and *Edaphobacter* were enriched in CK. *Burkholderia-Caballeronia-Paraburkholderia, Subgroup_2, Candidatus_Soilbactel, Magnetospirillaceae, Geobacter*, and *Pedosphaeraceae* were enriched in LHS treatment. While *Occallatibacter, Candidatus_Koribacter* and *Micropepsaceae* were enriched in HHS treatment. RDA ordination on genus level showed that CK and LHS treatments were significantly separated, indicating that LHS treatment caused significant changes in soil bacterial community structure (**Figure 6A**). The first two axes can jointly explain 85.7% of the variation degree of soil microbial community. Soil ammonium-nitrogen content, chlorophyll *a*+*b* content, and APX activity had a positively correlation to *Occallatibacter* and unclassified_Acidobacteriaceae_(Subgroup_1), while had a negatively correlation to *Burkholderia_Caballeronia*. MDA content, H_2_O_2_ content, and soluble sugar content were positively correlated with *Pedosphaeraceae*, while negatively correlated with *Acidothermus, Acidipila* and *Saccharimonadales*. Chlorophyll *a* content, chlorophyll *b* content, Pro content, and POD activity were positively correlated with *Conexibacter* and *Methylovirgula* (Figure 6A).

### 3.5. Response of Fungal Community

**Figure 5:**
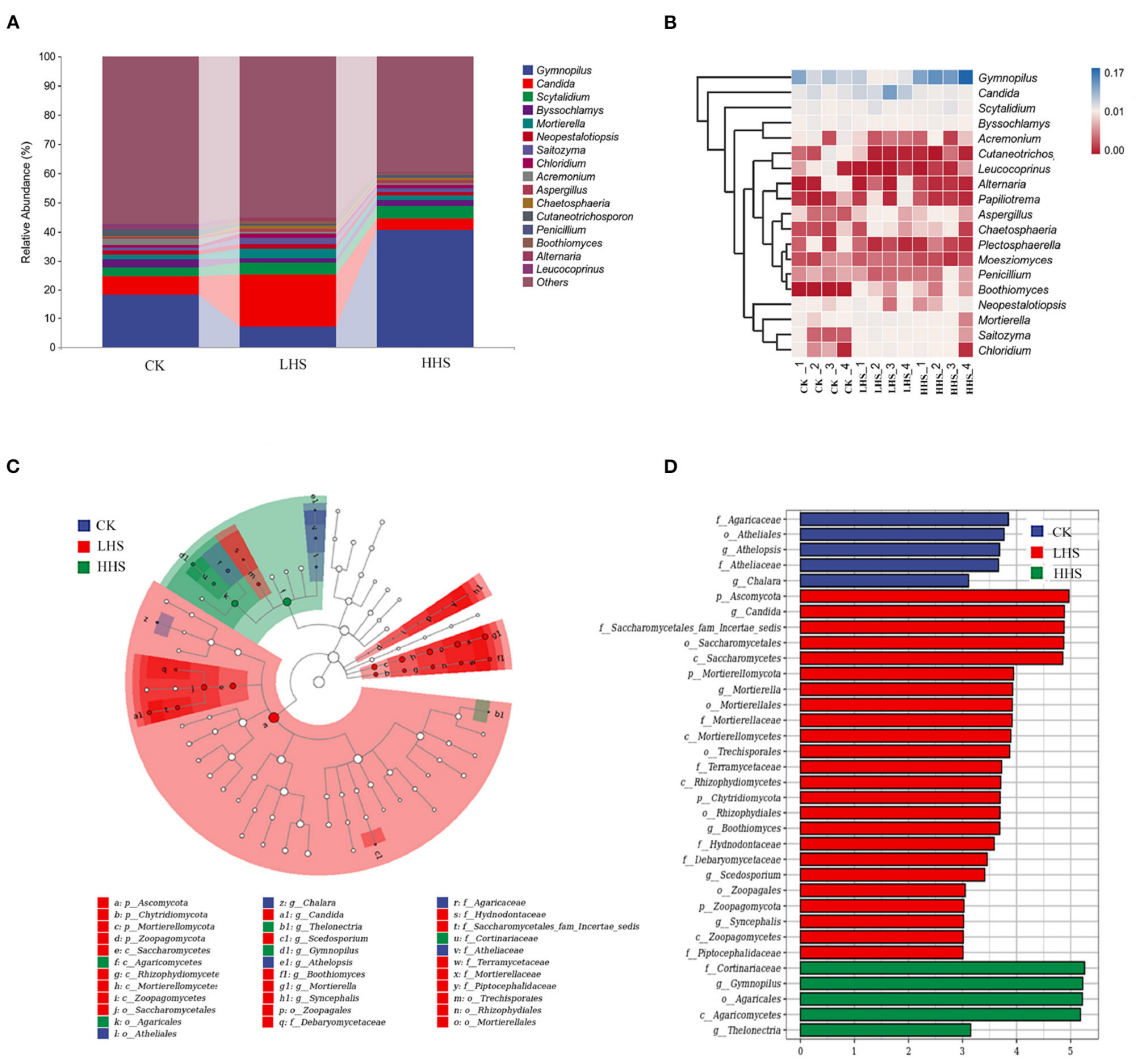
Comparison of fungal communities in CK, LHS and HHS treatments. **(A)** Fungal taxa at genus level. **(B)** Heatmap showing changes in microbial community in treatments at genus level. **(C)** Cladograms indicate the phylogenetic distribution of microbial lineages associated with three treatments. Each circle diameter is proportional to the taxon abundance. The strategy of multiclass analysis is strict (all classes differential). Circles represent phylogenetic levels from Kingdom to genus. **(D)** LDA bars indicate microbial groups within three treatments with LDA score higher than 3.0. The difference in abundant clades of each treatment are represented by colors as in cladograms, and the LDA scores of these clades indicate the degree of statistically and biologically difference. CK, control (25 /20°C); LHS, light heat stress (35/30°C); HHS, high heat stress (40/35°C); p, phylum; o, order; c, class; f, family; g, genus.

**Figure 6:**
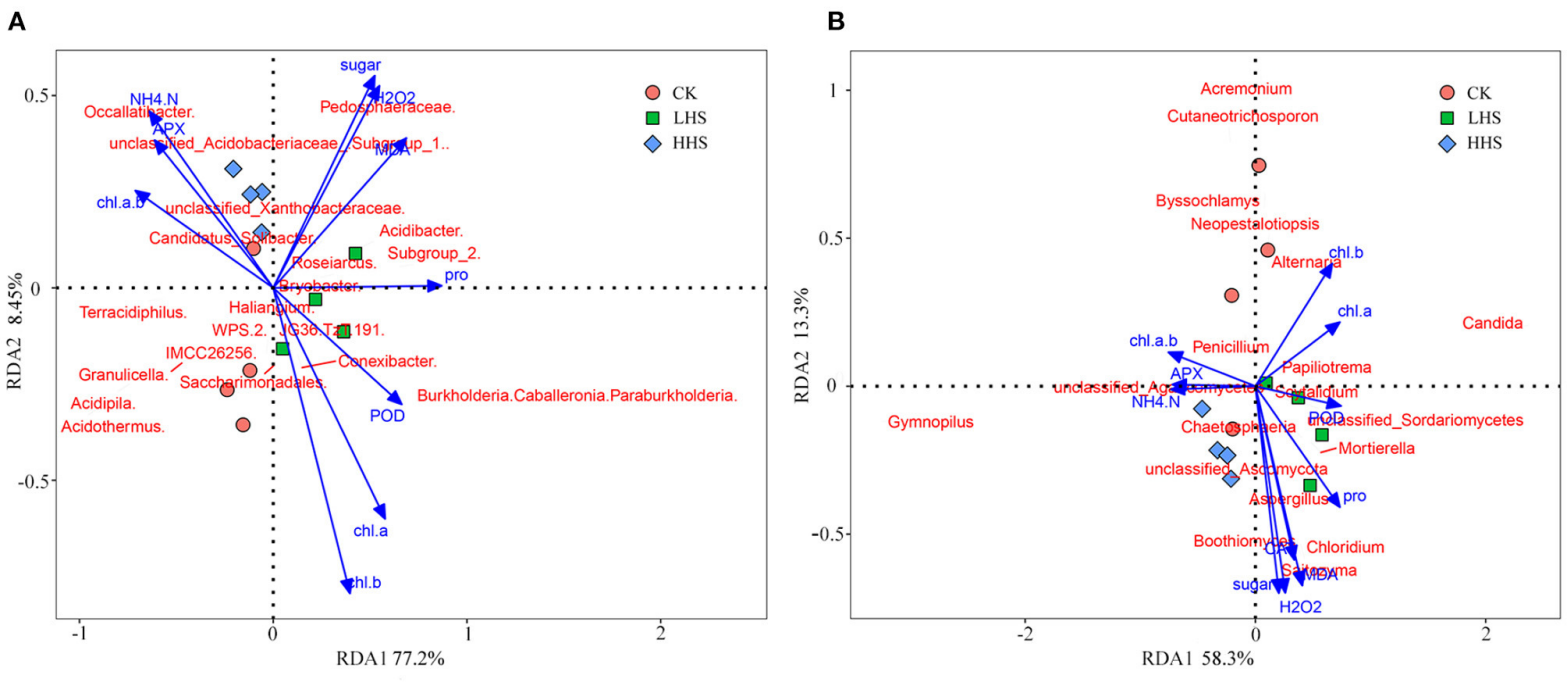
Redundancy analysis (RDA) of physiological characteristics, soil physicochemical properties, and soil dominant microbial communities of *Rhododendron simsii*, **(A)** RDA of each indicator with the top 1% of OTU values for dominant bacteria; **(B)** RDA of each indicator with the top 1% of OTU values for fungi. CK, control (25 /20°C); LHS, light heat stress (35 /30°C); HHS, high heat stress (40 /35°C); MDA, Content of malondialdehyde; H_2_O_2_ content of hydrogen peroxide; Pro content of proline; CAT, activity of catalase; POD, activity of peroxidase; APX, activity of ascorbate peroxidase; Chl *a*, content of chlorophyll *a*; Chl *b* content of chlorophyll *b*; Chl *a* + *b*, content of chlo-rophyll *a* + *b*; NH${}_{4}^{\;+}$−N, Content of soil ammonium-nitrogen.

A total of 848,406 validated fungal sequences were obtained after passing quality filtering. Sequence reading for per sample ranges from 60,086 to 8,094. We detected a total of 291 genera, and the total abundance of OTUs was higher in LHS treatment than that in the other treatments (Supplementary Figure 2). At the genus level, there were 19 genera with relative abundances higher than 1%, and the genus *Gymnopilus* was dominant in all treatments (relative abundance ranging from 4.2 to 23.0%), followed by genus *Candida* (relative abundance ranging from 2.1 to 11.3%) (Figure 5A and Supplementary Figure 2). Compared to CK, the abundance of *Candida, Mortierella* and *Boothiomyces* increased by 229.4, 108.5, and 10,658% in LHS treatment, respectively. Compared with CK, HHS treatment increased the abundance of *Gymnopilus* and *Papiliotrema* by 58.3 and 371.4%, respectively (Figure 5B). The sequencing covered a substantial portion of the bacterial diversity because the rarefaction curves tended to be plain (Supplementary Figure 3B). For fungal diversity indices, Shannon index was significantly higher under LHS treatment than that under HHS treatment, while for the Chao1 index was not significant differences among treatments (Supplementary Figure 4B). The fungal community structure in soil was analyzed. Cladogram showing the phylogenetic distribution of the fungal lineages in different treatment samples (Figure 5C). More fungal taxa were detected by LEfSe in LHS treatment (24 clades, 4 phylum, 4 classes, 5 orders, 6 families, and 5 genera) than that in other treatments. (Figure 5D). Significant differences appeared in the genus level. *Athelopsis* and *Chalara* were enriched in CK. *Candida, Mortierellales, Boothiomyces, Scedosporium*, and *Syncephalis* were enriched in LHS treatment. While *Gymnopilus* and *Thelonectria* were enriched in HHS treatment (Figure 5D).RDA analysis only showed the plant physiology and soil physicochemical factors, which is significantly correlated with soil fungal community (*P* < 0.05). The first two axes can jointly explain 71.6% of the variation degree of soil microbial community (Figure 6B). The results of RDA indicated that the content of MDA, H_2_O_2_, Pro, and soluble sugar had a positively correlation with the dominant fungal communities in soil, such as *Boothiomyces_macroporosum, Saitozyma_podzolica, Aspergillus_fumigatus*, and *Mortierella_ elongata*, while they had a negatively correlation with *Neopestalotiopsis_foedans, Byssochlamys_zollerniae*, and *Acremonium_furcatum*.

## 4. Discussion

### 4.1. Effect on Physiological Characteristics of *R. simsii* Under Heat Stress

Heat stress caused *R. simsii* to produce excessive MDA and H_2_O_2_ (Figure 2), which destroyed the integrity of the cell membrane. Both MDA and H_2_O_2_ levels increased under LHS treatment, which was consistent with the results of previous studies. Zhao et al. (2018) suggested that the excessive production of reactive oxygen species by plants under heat stress and the existence of antioxidant enzymes could not be removed in time, resulting in the accumulation of ROS. In the present study, both MDA and H_2_O_2_ were reduced under HHS treatment compared to CK, which possibly because plants cannot resist heat stress through self-regulation due to high heat stress temperature or prolonged pressure (Fang and Xiong, 2015).

In this study, compared to CK, LHS treatment significantly increased Pro content, while HHS treatment had no significant effect on Pro content. The soluble sugar content was significantly increased under both heat stress treatments, while soluble protein content did not change considerably under both heat stress treatments (Figure 3). Pro, soluble sugars, and soluble proteins are all essential osmotic regulators in plants, which can regulate cell water content and bulking pressure, to maintain the normal physiological functions of plants (Sezgin Muslu and Kadıoğlu, 2021). Similarly, some study reported that plant can maintain normal osmotic pressure under heat stress by accumulating large amounts of Pro and soluble sugars, thus alleviating heat stress (Veresoglou et al., 2012; Yang et al., 2020; Pei et al., 2021). And the soluble sugar content did not continue to rise under HHS treatment, probably due to the plant had reached its heat tolerance limit and could not continue to actively accumulate the soluble sugar (Zhao et al., 2018). In addition, it has been shown that soluble protein content may increase or decrease under heat stress. The decrease may be caused by heat disrupting the integrity of the plasma membrane and turning membrane protein into soluble protein (Ahammed et al., 2019).

Compared with CK, LHS treatment significantly increased CAT and POD activities, and HHS treatment significantly increased APX activity (Figure 3), the antioxidant system is also an important physiological mechanism to protect plants from heat stress by scavenging reactive oxygen radicals in plants. In this study, changes in APX activity corresponded with the results of chlorophyll content, which was remarkably able to scavenge H_2_O_2_ from chloroplasts, and the chlorophyll content can directly reflect the strength of photosynthetic performance in plants (Huihui et al., 2020). It is possible that chlorophyll content did not decrease under LHS treatment due to osmotic regulation and other protective enzymes. Compared with CK, HHS treatment decreased chlorophyll content, indicating that photosynthesis was inhibited by heat stress. The increase of APX activity in HHS treatment contributes to remove a large number of reactive species produced in the chloroplasts.

The changes in various physiological characteristics of *R. simsii* under heat stress varied depending on the test subjects or the time of treatment. However, when plants are subjected to excessive stress or prolonged stress, they cannot resist heat stress through self-regulation.

### 4.2. Effect of Heat Stress on Soil Physicochemical Properties

Compared to CK, LHS treatment significantly reduced soil ammonium-nitrogen and nitrate-nitrogen content, while HHS treatment significantly increased soil ammonium-nitrogen content and significantly reduced soil nitrate-nitrogen content. There were no significant changes in soil pH, WDOC, and MBN contents among treatments. Available nitrogen is an important nutrient that can be directly absorbed and utilized by plant roots in the soil, which also plays a very important role in regulating soil enzyme activity, plant metabolism, and soil nutrient accumulation and transformation (Wang et al., 2019). The decrease of ammonium-nitrogen and nitrate-nitrogen content under LHS treatment was possibly because LHS accelerated the uptake and utilization of nitrogen by plants for synthesizing substances such as osmoregulation substances and protective enzyme (Luo et al., 2021). The change in available nitrogen under HHS treatment may be caused by the inhibition of the conversion of ammonium-nitrogen to nitrate-nitrogen at 40°C (Wen et al., 2019), or by the internal adjustment of the rate of nitrogen and oxygen utilization in the soil ecosystem to adapt to the external environment (Hermann et al., 2021). In this study, the insignificant change in soil pH value and WDOC content between CK and the two treatments may be due to that this study was treated for a short period of time and no significant change had occurred (Ashraf et al., 2019). In addition, it has been shown high temperature reduced MBC and MBN content, probably due to the high temperature could reduce soil microbial activity (Xu and Yuan, 2017), which is consistent with the change of MBN content in this study. Therefore, soil available nitrogen content was sensitive to heat stress in a short time, WDOC, MBNand pH value did not change significantly.

### 4.3. Effect of Heat Stress on Soil Bacterial Community and Its Association With Physiological Characteristics of *R. simsii* and Soil Physicochemical Properties

In this study, we found that LHS treatment significant changed bacterial community structure (Figure 4A). For example, LHS treatment remarkably reduced *Acidothermus* abundance, which is inconsistent with previous studies (Fierer et al., 2007; Zhang et al., 2013), as *Acidothermus* is thermophilic, but is consistent with Riah-Anglet et al. (2015). We also found LHS treatment significantly increased the abundance of BCP, possibly because BCP can promote plant growth and help plants resist heat stress (Figure 4B). However, the abundance of BCP did not change significantly under HHS treatment, which may be due to the high temperature and the mutual benefit between plant and bacterial community. In addition, the abundance of *Pseudomonas* increased under heat treatment in this study, which is consistent with previous studies (Ali et al., 2009, Ali et al., 2011, Ashraf et al., 2019), this genus of bacteria is considered to be a beneficial microorganism that can alleviate heat stress of plants (Shekhawat et al., 2022).

In this study, MDA and H_2_O_2_ content of *R. simsii* were significantly negative correlated with *Acidothermus* (Figure 6A), which also known as Acidophilus thermophilus, prefers hot, acidic soil conditions. It was found that the increase in *Acidothermus* abundance means an increase in soil organic matter content and soil fertility, which are beneficial to plant resistance to stress (Ren et al., 2021; Xia et al., 2022). Under normal conditions, soil microorganisms and plants benefit from each other. However, if subjected to a stressful environment, soil microorganisms may compete with plants for nutrients in the soil. Besides, the results of RDA showed that soil ammonium-nitrogen content, APX activity, and chlorophyll *a*+*b* content has a significant positive correlation with *Occallatibacter* and unclassified_Acidobacteriaceae_(Subgroup_1), and has a negatively correlation with BCP (Figure 6A). Acidobacteriaceae, mostly acidophilic bacteria, are a common group of soil bacteria that play an important role in soil ecology, degrade cellulose, participate in iron cycling, and single-carbon metabolism (Zhou et al., 2020). In this study, the positively correlation between Acidophilidae and ammonium-nitrogen content suggested that Acidophilidae bacteria can enhance soil material cycle metabolism and increase soil ammonium-nitrogen content. Plants can absorb and use available nitrogen in the soil through the root system, which also plays a vital role in nutrient accumulation and transformation, soil enzyme activity and plant metabolism (Gu et al., 2019). Furthermore, microbial communities such as symbiotic nitrogen-fixing bacteria, mycorrhizal fungi and rhizosphere growth-promoting bacteria can form symbiotic relationships with plant roots, which in turn affects the uptake of soil nutrients by plants (Mu et al., 2021). In addition, soil microorganisms decompose organic nitrogen and release inorganic nitrogen through their metabolic processes or mineralization (Myrold, 2021). As nitrogen is an important component of chlorophyll and most enzymes, the increase of soil ammonium-nitrogen content affects the APX activity and chlorophyll *a*+*b* content of *R. simsii*. In this study, the reduction in available nitrogen content under LHS treatment may be caused by the internal adjustment of the nitrogen and oxygen utilization rate by the soil internal ecosystem to adapt to the external environment (Veresoglou et al., 2019). However, the reduction of nitrate-nitrogen content under HHS treatment may be since the high temperature of 40°C inhibited the activity of some soil microorganisms or changed their abundance, thus slowing down the conversion of ammonium-nitrogen to nitrate-nitrogen. In short, combined with our results, beneficial bacteria such as BCP group can directly or indirectly regulate the regulation of heat stress in plants under high temperature stress, but their complex regulation mechanism needs to be further studied.

### 4.4. Effect of Heat Stress on Soil Fungal Community and Its Association With Physiological Characteristics of *R. simsii* and Soil Physicochemical Properties

The effects of soil fungal community and on soil nutrients and plant physiological characteristics are similar to bacterial community. In this study, HHS treatment significantly reduced soil fungal diversity, but had no effect on bacterial diversity, which may be due to the fact that fungi are more sensitive to heat stress than bacteria (Rinnan et al., 2007). Furthermore, we found that LHS treatment significantly changedthe fungal structures in soil (Figure 5B). LHS treatment significantly increased the abundance of *Candida, Mortierella, Saitozyma*, and *Chloridium*, while significantly decreased the abundance of *Acremonium* and *Cutaneotrichosporon*, possibly because of the different sensitivity of these fungal community to heat stress. *Cutaneotrichosporon* was observed to have the capacity to generate higher saturated and monounsaturated fatty acid concentrations can enhance cold and disease resistance (Zahid et al., 2021). The abundance of *Mortierella* increased significantly under LHS treatment, which provided protected to plants. However, the abundance of *Mortierella* decreased significantly under HHS treatment. Thus, we suggest that HHS treatment induces the death of heat-sensitive species, reduces competition, promotes the spread of beneficial species, and ultimately leads to better adaptation of microbial communities to the environment (Riah-Anglet et al., 2015). However, as fungi were more sensitive to HHS treatment, the community structure and function of fungi were less stable than that of LHS.

The results of this study showed that MDA, H_2_O_2_, soluble sugar content, and CAT activity was positively correlated with *Saitozyma, Chloridium*, and *Boothiomyces*, while negatively correlated with *Acremonium* and *Cutaneotrichosporon* (Figure 6B). *Chloridium* is an endophytic fungus that derives its support and nutrients from plant roots and, in turn, produces possible antimicrobial agents to protect plants from environmental threats (Vardharajula et al., 2017). Soil ammonium-nitrogen, chlorophyll content and APX activity were positively correlated with unclassified_Agaricomycetes, while negatively correlated with Candida. Some of the fungi in *Mortierella* can participate in soil nutrient transformation and promote plant growth. Hu et al. (2022) concluded that *Mortierella* could significantly increase soil soluble organic carbon, available nitrogen and related enzyme activities, and interoperate with microorganisms. It has been shown that *Candida* has the ability to dissolve inorganic phosphorus and produce IAA, and has biological control and soil remediation functions, which can promote plant germination and *R. simsii* growth as well as increase resistance to adversity stresses (Petkova et al., 2022). This is generally consistent with the results of the present study. Pro content was positively correlated with *Mortierella*. Some of the fungi in *Mortierella* can participate in soil nutrient transformation and promote plant growth. Hu et al. (2022) concluded that *Mortierella* could significantly increase soil soluble organic carbon, available nitrogen and related enzyme activities, and interoperate with microorganisms. Thus, soil microorganisms play an important role in plant resistance to heat stress.

## 5. Conclusion

In this study, we found that plant physiological characteristics, soil ammonium-nitrogen content is closely related to soil microbial community under heat stress. The results showed that MDA, H_2_O_2_, antioxidant enzyme, chlorophyll and other physiological indexes of *R. simsii* were closely related to soil ammonium-nitrogen content under heat stress. We also showed that the LHS treatment had a greater effect on the changes of bacterial and fungal communities than HHS treatment. The LHS treatment significantly altered soil microbial community structure compared to CK, and some of the bacterial (*Burkholderia-Caballeronia-Paraburkholderia*) and fungal (*Candida, Mortierella* and *Boothiomyces*) communities may help *R. simsii* resist heat stress. We concluded that *R. simsii* may cooperate with soil microbial communities to obtain nutrients from the soil to help them resist heat stress when heat stress is light. However, the mutually beneficial relationship between *R. simsii* and microorganisms under HHS seems to be no obvious, and the reasons for this need to be further studied.

## Data Availability Statement

The raw data supporting the conclusions of this article will be made available by the authors, without undue reservation. The bacterial isolates and fungi were provided and available at The National Center for Biotechnology Information Sequence Read Archive Database with Accession Codes PRJNA853115 and PRJNA852402, respectively.

## Author Contributions

LL and XT: writing—original draft preparation. YL: investigation. LZ, SL, WL, and LG: review and editing. SW and YZ: methodology. LL, XiaoC, and LW: formal analysis. XiangC: validation. LG: funding acquisition. All authors have read and agreed to the published version of the manuscript.

## Funding

This research was funded by The Hainan Provincial Natural Science Foundation of China, grant numbers (2019RC111 and 320RC470), and Priming Scientific Research Foundation of Hainan University (KYQD(ZR)1982).

## Conflict of Interest

The authors declare that the research was conducted in the absence of any commercial or financial relationships that could be construed as a potential conflict of interest.

## Publisher's Note

All claims expressed in this article are solely those of the authors and do not necessarily represent those of their affiliated organizations, or those of the publisher, the editors and the reviewers. Any product that may be evaluated in this article, or claim that may be made by its manufacturer, is not guaranteed or endorsed by the publisher.
