# Supplementary figures and images for: Changes and Correlation Between Physiological Characteristics of Rhododendron simsii and Soil Microbial Communities Under Heat Stress
Source: Front Plant Sci. 2022 Jul 22;13:950947. doi: 10.3389/fpls.2022.950947 (PMC9355081; doi:10.3389/fpls.2022.950947)

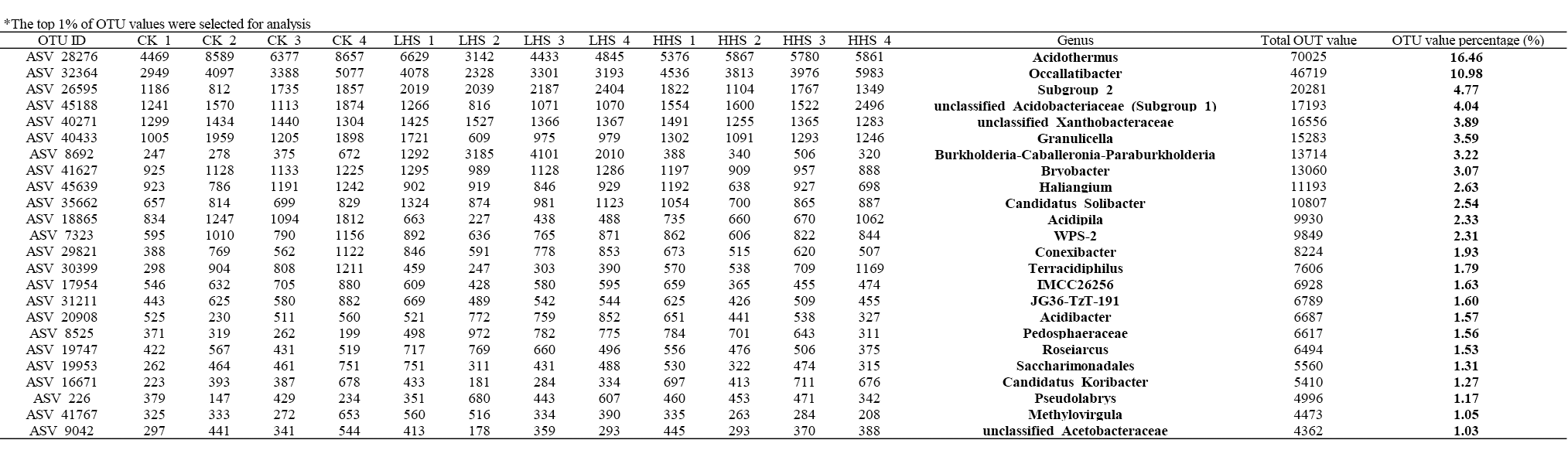

Supplement: Supplementary file 1 [file Image_1.TIF]

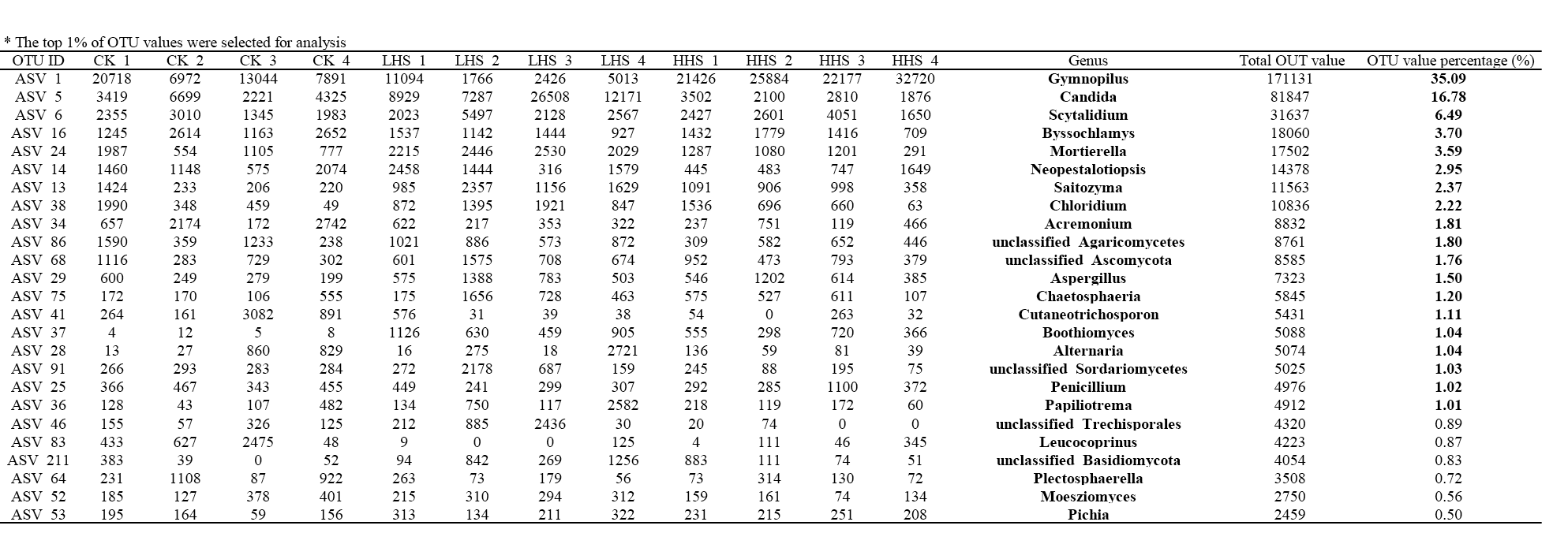

Supplement: Supplementary file 2 [file Image_2.TIF]

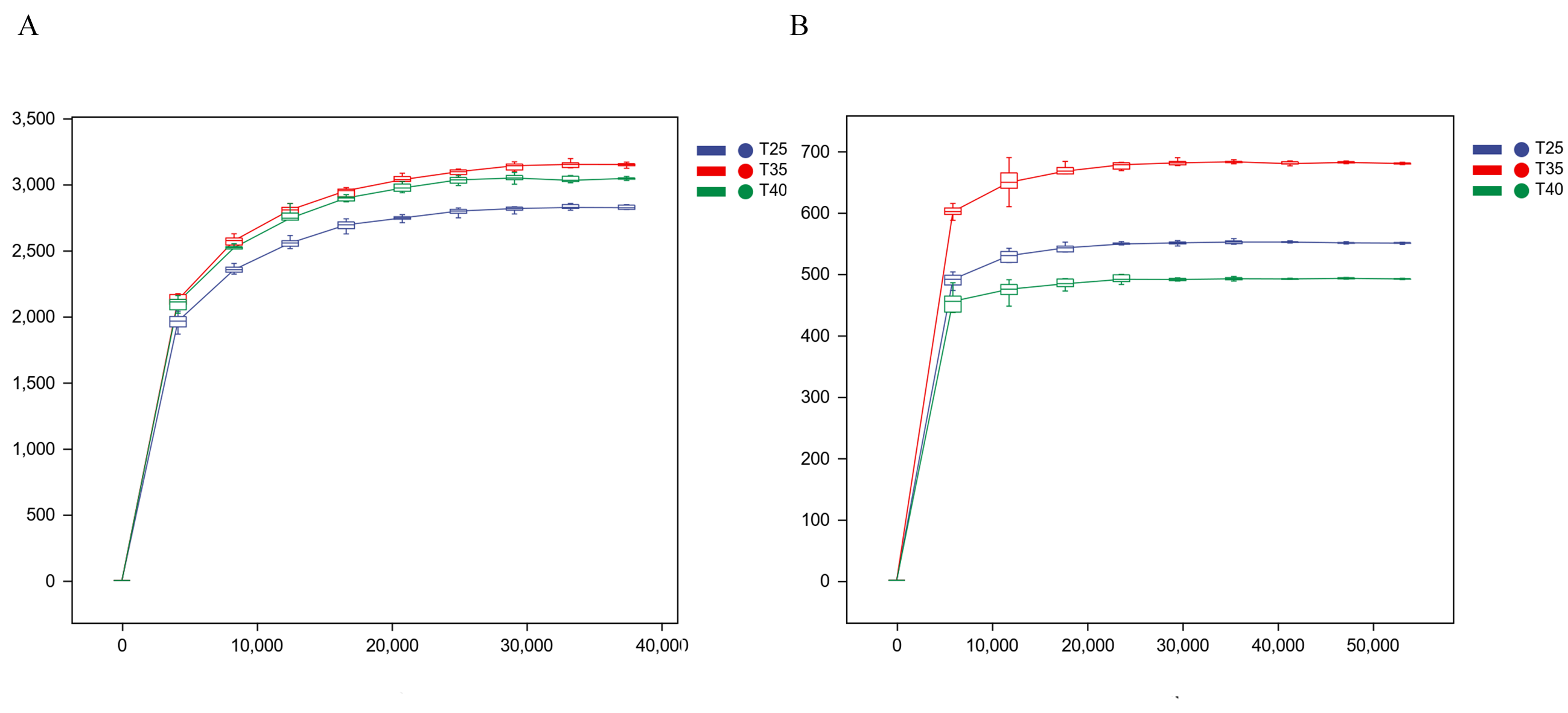

Supplement: Supplementary file 3 [file Image_3.TIF]

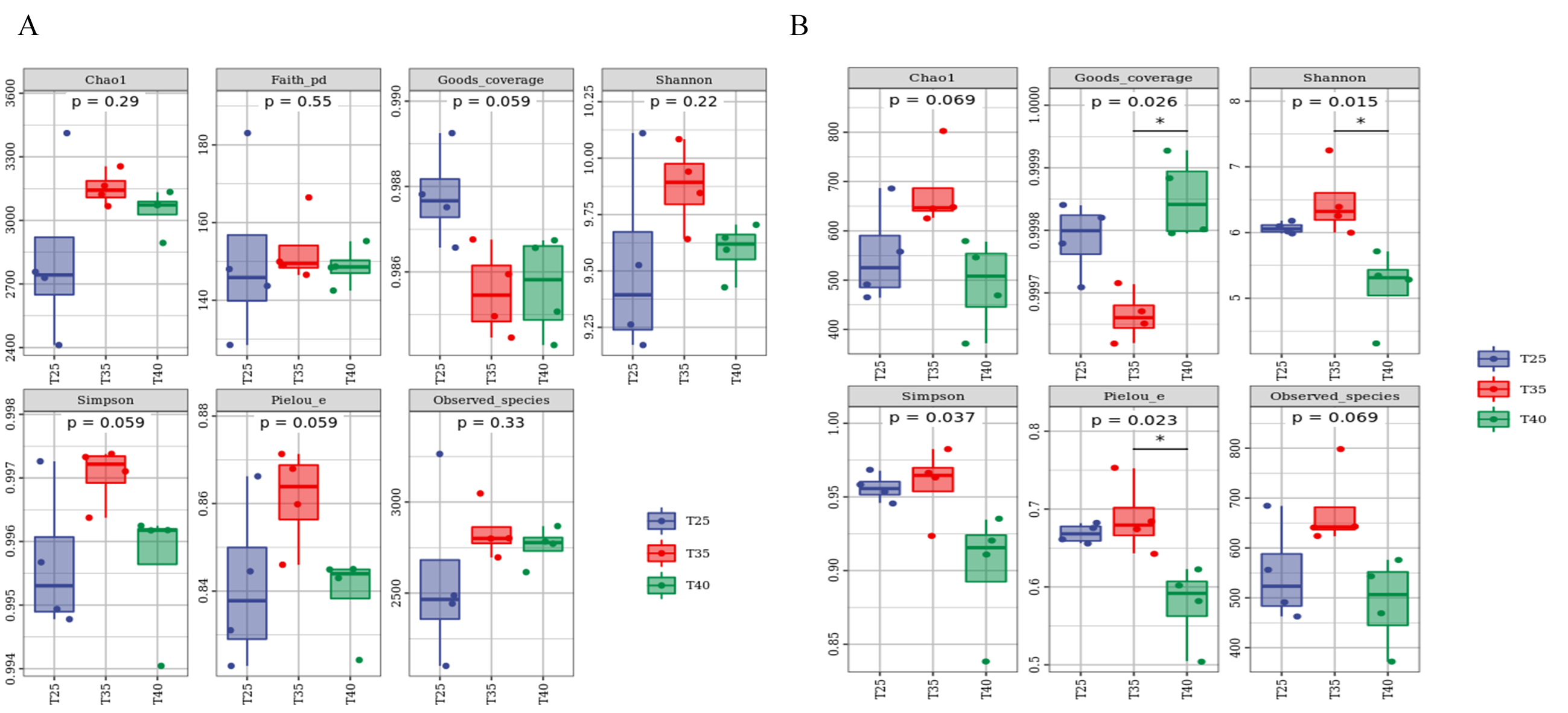

Supplement: Supplementary file 4 [file Image_4.TIF]
